# Identification and Analysis of Candidate Genes Associated with Maize Fusarium Cob Resistance Using Next-Generation Sequencing Technology

**DOI:** 10.3390/ijms242316712

**Published:** 2023-11-24

**Authors:** Aleksandra Sobiech, Agnieszka Tomkowiak, Jan Bocianowski, Grażyna Szymańska, Bartosz Nowak, Maciej Lenort

**Affiliations:** 1 Department of Genetics and Plant Breeding, Poznań University of Life Sciences, Dojazd 11, 60-632 Poznań, Poland; aleksandra.sobiech@up.poznan.pl (A.S.); macieklenort@gmail.com (M.L.); 2 Department of Mathematical and Statistical Methods, Poznań University of Life Sciences, Wojska Polskiego 28, 60-637 Poznań, Poland; jan.bocianowski@up.poznan.pl; 3 Department of Agronomy, Poznań University of Life Sciences, Dojazd 11, 60-632 Poznań, Poland; grazyna.szymanska@up.poznan.pl; 4 Smolice Plant Breeding Sp. Z o.o. IHAR Group, Smolice 146, 63-740 Kobylin, Poland; nowak@hrsmolice.pl

**Keywords:** maize, NGS, association mapping, fusarium, SNP markers, SilicoDArT markers

## Abstract

The pressure to reduce mineral fertilization and the amount of pesticides used has become a factor limiting production growth, as has the elimination of many crop protection chemicals from the market. A key condition for this to be an effective form of protection is the use of varieties with higher levels of resistance. The most effective and fastest way to assist in the selection and control of pathogens is the conducting of genome-wide association studies. These are useful tools for identifying candidate genes, especially when combined with QTL mapping to map and validate loci for quantitative traits. The aim of this study was to identify new markers coupled to genes that determine maize plant resistance to fusarium head blight through the use of next-generation sequencing, association and physical mapping, and to optimize diagnostic procedures to identify selected molecular markers coupled to plant resistance to this fungal disease. As a result of field experiments and molecular analyses, molecular markers coupled to potential genes for resistance to maize ear fusariosis were selected. The newly selected markers were tested against reference genotypes. As a result of the analyses, it was found that two markers (11801 and 20607) out of the ten that were tested differentiated between susceptible and resistant genotypes. Marker number 11801 proved to be the most effective, with a specious product of 237 bp appearing for genotypes 1, 3, 5, 9 and 10. These genotypes were characterized by a field resistance of 4–6 on the 9° scale (1 being susceptible, 9 being resistant) and for all genotypes except 16 and 20, which were characterized by a field resistance of 9. In the next step, this marker will be tested on a wider population of extreme genotypes in order to use it for the preliminary selection of fusarium-resistant genotypes, and the phosphoenolpyruvate carboxylase kinase 1 gene coupled to it will be subjected to expression analysis.

## 1. Introduction

The use of biotechnological tools in plant resistance breeding is currently a priority because the enormous pressure of pathogens causes a significant decline in crop yields. Due to such serious losses, the breeding company and agricultural sectors of the world’s countries are striving to increase and sustainably produce food [1]. Unfortunately, the breeding of new, fusarium-resistant varieties has its limitations, both technological and economic, due to the ratio of the prices of production inputs to the price of cereals [2]. Intensification of the breeding of resistant varieties should be profitable; however, many times it is unfortunately the case that the increase in additional expenditures on fertilizers and protection products is not offset by an increase in yields [3].

The principle by which integrated plant protection is applied by all agricultural producers in the European Union has been in effect since 1 January 2014 and was put in place to protect the environment and to ensure the safety of human health. One of the general principles of integrated plant protection is to prevent the occurrence of harmful organisms or limit their negative impact, which should be achieved with, among other strategies, the use of resistant or tolerant varieties [4,5].

A key factor allowing for this to become an effective form of protection is the use of varieties with higher levels of resistance. The burden of responsibility for further production growth is thus increasingly shifting from agrotechnology to breeding, which, as we have seen, is able to cope effectively [6]. Consequently, newer tools are being developed with which to guarantee greater efficiency in selection [7].

The selection methods presently in use have been enriched by advances in molecular biology and statistical models that allow the identification of markers for individual traits that are the result of single genes and for markers that are determined by multiple quantitative trait loci (QTLs) that explain the phenotypic variation of a trait to varying degrees [2,8]. Progress in crop production depends on the development of cultivation and plant breeding methods based on information about plant properties and their behavior under different growing and environmental conditions. Scientists have been conducting research projects dedicated to learning about these properties through observations at the genome, transcriptome, proteome, metabolome and phenome levels [9]. It is important that the information obtained in the research be collected in a coordinated manner and in public databases accessible to breeders and other interested parties [10].

As a result of climate change and the intensification of international trade, pathogen pressure is also increasing. Maize plants are most often infected by pathogenic fungi, including *Fusarium graminearum* and *Setosphaeria turcica*, causing severe yield losses [11,12]. Pathogenic fungi are naturally present in the soil and cannot be eliminated, so it is important to control them in order to reduce the production of mycotoxins that are produced by fungi [13]. Attention should therefore be focused on the nutritional and structural needs of both the plant and its microbiome. Several studies have shown that soil fertilization has had a positive effect on plant tolerance to pathogenic fungi, such as in instances where fertilizers containing either zinc or potassium phosphite have been used [14,15,16].

However, the most effective and fastest way to aid in pathogen selection and control are transcriptomic and genome-wide association studies (GWAS). These are useful tools for identifying candidate genes, especially when combined with QTL mapping to map and validate loci for quantitative traits [17]. Combining these methods allows for the overcoming of their respective limitations [18]. As an alternative to classical plant breeding methods, precision genetic engineering based on genome editing technologies can play a key role in accessing genetic resources. These resources can be used to increase plant resistance to disease by targeting appropriate genetically conditioned plant defense mechanisms [19].

The aim of the study was to identify new markers coupled to genes determining resistance of maize plants to fusarium through the use of next-generation sequencing, association and physical mapping, and to optimize diagnostic procedures with which to identify the selected molecular markers coupled to plant resistance to this fungal disease.

## 2. Results

### 2.1. Field Experiment

**Figure 1:**
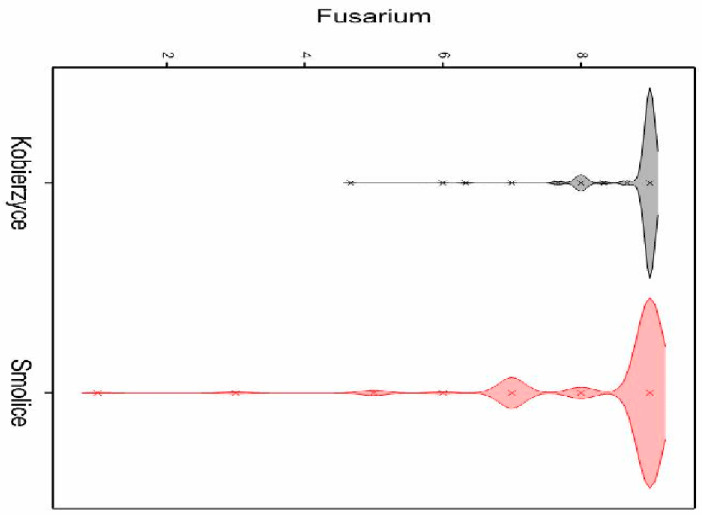
Density plot of the distribution of *Fusarium* infestation of surveyed lines in both localities.

In order to examine the distribution of the analyzed variable in both localities (Smolice and Kobierzyce), a density plot was made (Figure 1). As can be seen in the attached graph, the distribution of the analyzed variable differed between Smolice and Kobierzyce. In Smolice, the majority of plants were infected by fusarium in the range of 6.5 to 9 on a 9 degree scale (9 being resistant and 1 being susceptible), while in Kobierzyce it ranged from 8 to 9 (Figure 1).

### 2.2. Phenotyping

Analysis of variance indicated that the main effects of genotype, location, and genotype × location interaction were significant for the observed degree of fusarium infection of maize plants (Table 1).

**Figure 2:**
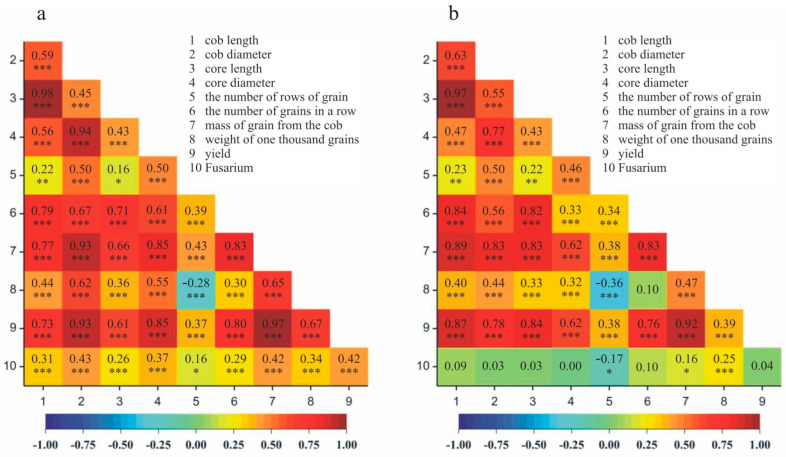
Heatmap showing Pearson correlation coefficients between the degree of *Fusarium* infestation of plants and yield and yield structure traits in s in (**a**) Kobierzyce and (**b**) Smolice. * *p* < 0.05; ** *p* < 0.01; *** *p* < 0.001.

For both localities (Smolice and Kobierzyce), correlations were analyzed between the degree of plant *Fusarium* infestation and the size of yield structure traits and yield per plot. It was shown that, in Smolice, the degree of plant *Fusarium* infestation was most positively correlated with the weight of grain per cob (*r* = 0.16)—the lower the infestation, the higher the weight of grain per cob—and the number of grains per row (*r* = 0.10) (Figure 2a). In Kobierzyce, the degree of plant *Fusarium* infestation was most positively correlated with yield per plot (*r* = 0.42)—the lower the infestation the higher the yield—as well as with grain weight per cob (*r* = 0.41) and weight of one thousand grains (*r* = 0.33) (Figure 2b).

### 2.3. Genotyping

**Figure 3:**
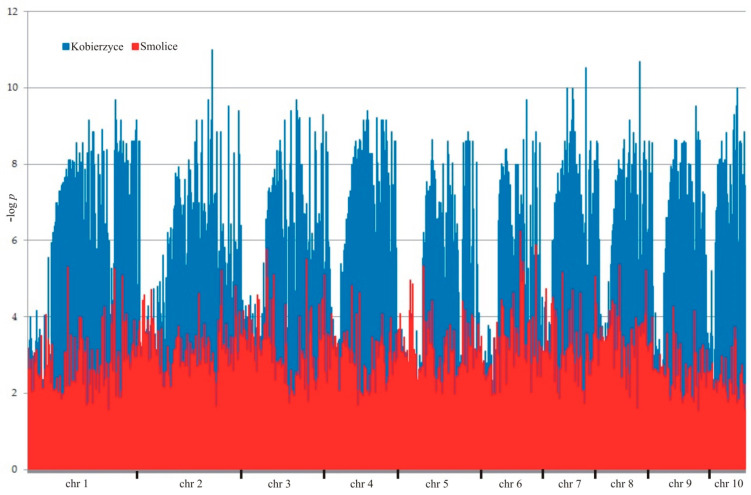
Manhattan plot for maize plant resistance to fusarium in Kobierzyce (blue) and Smolice (red).

Next-generation sequencing obtained a total of 92,614 molecular markers (60,436 SilicoDArT and 32,178 SNPs). To determine the significance of the identified markers, MAF > 0.25 and the number of missing observations <10% were used. This way, 32,900 (26,234 DArTs and 6666 SNPs) markers were obtained and used for association mapping (Figure 3). Significantly associated (selected at *p* < 0.001 with correction for Benjamini–Hochberg multiple testing) with fusarium resistance were 6816 SNP and silico DArT molecular markers (5985—Kobierzyce, 531—Smolice) (Table 2). In order to narrow down the number of markers for physical mapping, 10 markers that were significant in both localities (Kobierzyce and Smolice) were selected from among all of them. These markers have the highest LOD values, making them the best tools for the breeding process.

**Figure 4:**
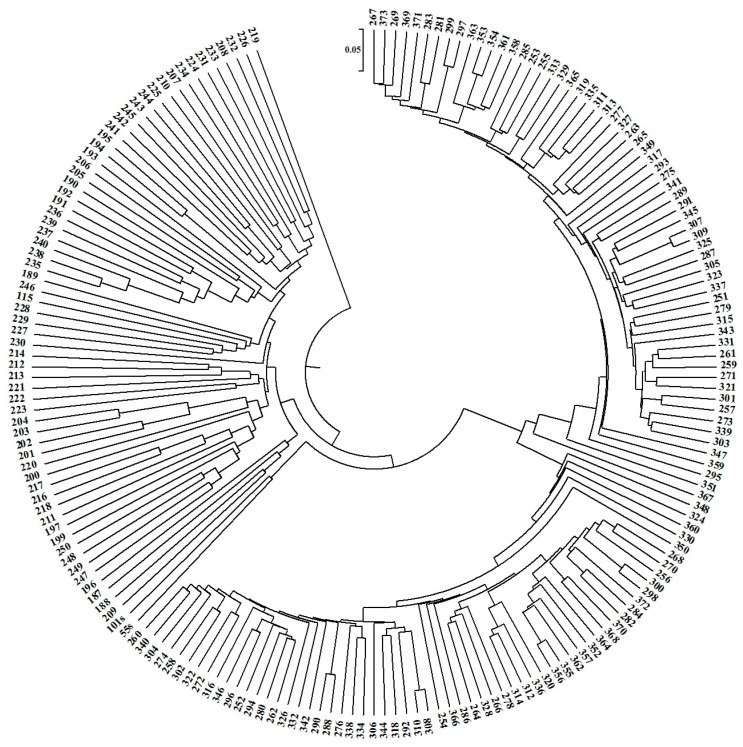
Dendrogram showing the genetic similarity between the analyzed genotypes. The numbers on the dendrogram indicate the individual genotypes.

Based on the identified SNP and SilicoDArT molecular markers, a dendrogram of genetic similarity between the 188 analyzed genotypes was made (Figure 4). The dendrogram very clearly shows two distinct groups of similarity. The first group includes 65 inbred lines from plant breeding in Kobierzyce, while the second group includes 122 analyzed hybrids and 1 inbred line. Such an ideal grouping demonstrates the usefulness of SNP and silico DArT markers for grouping genotypes in terms of genetic similarity.

Of the 32,900 (26,234 DArTs and 6666 SNPs) markers selected for association mapping, 10 were selected that were significantly associated with plant resistance to fusarium in both locations—Kobierzyce and Smolice (Table 3). An attempt was also made to determine the location of the selected DArT (9) and SNP (1) markers. The next step was to design primers that served to identify the 10 selected markers. The sequences of the primers are shown in Table 4.

### 2.4. Identification of New Molecular Markers Associated with Fusarium Resistance in Maize Plants Using Polymerase Chain Reaction (PCR)

The new selected markers were tested on 20 reference genotypes from plant breeding in Smolice and Kobierzyce. On agarose gels, the genotypes were arranged in the following order: from 1 to 10 are genotypes susceptible to fusarium in field conditions, while from 11 to 20 are genotypes resistant to fusarium in field conditions. The analyses found that 2 markers (11801 and 20607) of the 10 tested differentiated between susceptible and resistant genotypes. For the remaining eight markers, monomorphic amplification products were observed on agarose gels with sizes consistent with the expected values in Table 4.

#### 2.4.1. Marker 11801 (237 bp Product)

**Figure 5:**
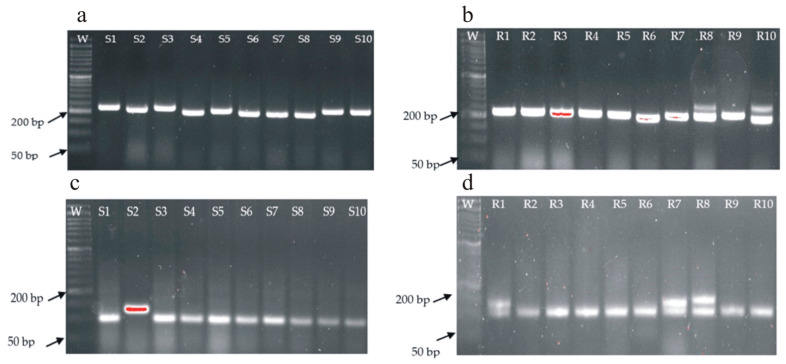
Electrophorogram illustrating: (**a**) 237 bp amplification products specific to the SilicoDArT 11801 marker—susceptible genotypes, (**b**) 237 bp amplification products specific to the SilicoDArT 11801 marker—resistant genotypes, (**c**) 137 bp amplification products specific to the SilicoDArT 20607 marker—susceptible genotypes, and (**d**) 137 bp amplification products specific to the SilicoDArT 20607 marker—resistant genotypes.

Analysis of marker number 11801 showed that a specific product of 237 bp appeared for genotypes: S1, S3, S5, S9 and S10. These genotypes were characterized by a field resistance level of 4–6 on the 9° scale (1 being susceptible and 9 being resistant) and for all genotypes except R6 and R10, which were characterized by a field resistance level of 9. This marker and the gene linked to it require further analysis to be used for the initial selection of fusarium-resistant genotypes (Figure 5a,b).

#### 2.4.2. Marker 20607 (137 bp Product)

Analysis of marker number 20607 showed that a specific product of 137 bp appeared for genotype number S2, which had a field resistance level of 6 on the 9° scale (1 being susceptible and 9 being resistant), and for genotypes R1, R7 and R8, which had a field resistance level of 9. This marker, like marker 11801, needs to be tested on a larger population of extreme genotypes so that a decision can be made on its use for the initial selection of fusarium-resistant genotypes (Figure 5c,d).

## 3. Discussion

In the era of integrated pest management, which has been in force for several years, as well as the successive reduction in the amount of pesticide active substances available, it is particularly important to select for cultivation varieties that are less susceptible to disease. In the case of maize, the pathogens that have been causing huge yield losses for years are *Fusarium* fungi.

Fusarium is a disease that occurs in many parts of the world and is considered a major factor affecting the size and quality of the grain yield obtained. The disease is caused by fungi belonging to the genus *Fusarium*, mainly *F. culmorum*, *F. graminearum*, and *F. verticillioides* [20]. *Fusarium* spp. infect maize grain and other above-ground parts, leading to significant yield losses and a deterioration of maize grain quality [21]. *Fusarium graminearum* causes maize cob rot and is a major cause of yield losses in Canada [22], China [23], and, in Europe, Italy [20,24]. Weather conditions are more of a factor influencing *Fusarium* infestation of cereal grains than varying agricultural systems, a theory that is supported by the research of Champeil et al. [25]. Assessment of fusarium risk is based on weather conditions and is undertaken from flowering to milk maturity of the grain [26]. The emergence of the European corn borer (*Ostrinia nubilalis*), which feeds on maize, also increases the susceptibility of maize to fusarium infection.

In the present experiment, in addition to in-depth molecular analyses, observations were made on the degree of infestation of maize plants by *Fusarium* fungi. Analysis of variance showed significant variation in fusarium resistance between genotypes and statistically significant variation in this trait between localities where the field experiment was established. For both localities (Smolice, Kobierzyce), correlations were analyzed between the degree of plant *Fusarium* infestation and the size of yield structure traits and yield per plot. It was shown that, in Smolice, the degree of plant *Fusarium* infestation was most positively correlated with the weight of grain per cob (0.16)—the lower the infestation the higher the weight of grain per cob, and the number of grains per row (0.10). In Kobierzyce, the degree of plant *Fusarium* infestation was most positively correlated with yield per plot (0.42)—the lower the infestation the higher the yield—as well as with grain weight per cob (0.41) and weight of one thousand grains (0.33). Maize resistance to *Fusarium* spp. is conditioned by multiple genes and the heredity of this trait is very complex [27,28,29]. Understanding the genetic mechanisms controlling resistance to fungal diseases facilitates maize breeding. As early as 1994, Hoenisch and Davis [30] showed that physical factors such as pericarp thickness are involved in resistance to cob rot. Bily et al. [31] and Sampietro et al. [32] have suggested that chemical factors such as phenolic compounds are involved in resistance processes. Many authors believe that plant resistance to fusarium is the result of interactions between genes. Opinions on this topic are divided because some researchers believe that these interactions are additive and others epistatic or have a large role in the dominance effect [4,29,33,34,35]. Zhou et al. [36], based on a genetic linkage map constructed using 1868 markers, identified 11 QTLs, including 5 stable QTLs. Collaborative analysis of multiple environments and epistatic interactions revealed six additive and six epistatic QTLs, respectively. The authors showed that many QTL with small effects had an impact on fusarium resistance. These were both additive and epistatic effects. The QTL qGER4.09, with the largest effect identified and validated using 588 F2 individuals, was linked to genomic regions associated with fusarium ear rot and aspergillus ear rot. This suggests that the described locus likely confers resistance to multiple pathogens and could potentially be used in maize variety breeding to improve resistance to cob rot diseases. Exploiting existing sources of resistance in breeding programs requires a thorough understanding of the genetic basis of maize resistance to fusarium.

In view of the above, the aim of the present study was to identify new markers coupled to genes determining maize plant resistance to fusarium through the use of next-generation sequencing, association and physical mapping, and to optimize diagnostic procedures to identify the selected molecular markers coupled to plant resistance to this fungal disease.

In our own research, DArTseq technology developed at Diversity Arrays Technology in Australia was used for sequencing. As of now, the most common NGS techniques include pyrosequencing 454 [37], the Solex technique (Illumina, San Diego, CA, USA), the SOLiD platform (Life Technologies Corporation, Carlsbad, CA, USA), Polonator (Harvard University, Cambridge, MA, USA), and the HeliScope Single Molecule Sequencer (Helicos BioSciences, Cambridge, MA, USA). These technologies provide inexpensive whole-genome sequence reads using methods such as chromatin immunoprecipitation, mutation mapping, polymorphism detection, and detection of non-coding RNA sequences [38]. Sequencing methods, such as restriction site associated DNA (RAD) [39], multiplexed shotgun genotyping (MSG) [40] and BSRSEq (RNA-Seq) [41], allow for the identification of a large number of markers and for the accurate testing of multiple loci in a small number of samples. The Illumina method has given rise to the development of GBS [42] and DarTseq procedures [43].

In the present study, a total of 92,614 molecular markers (60,436 SilicoDArT and 32,178 SNPs) were obtained using the DArTseq technique. To determine the usefulness of the identified markers, MAF > 0.25 and the number of missing observations <10% were used. This resulted in 32,900 (26,234 DArTs and 6666 SNPs) markers, which were used for association mapping.

Genome-wide association studies (GWAS) are a useful tool for identifying candidate genes coupled to quantitative traits. Zila et al. [8,44], using GWAS, identified SNP markers associated with maize fusarium resistance. The authors identified 10 SNP markers significantly associated with resistance to this pathogen. Zila et al. [44] identified SNP markers associated with defense response in and adjacent to the sequences of five genes. These areas had not previously been correlated with disease resistance but were attributed to a function related to the programmed cell death pathway. Through GWAS analyses, many authors have identified QTL regions associated with plant resistance to biotic stresses. De Jong et al. [45] identified these regions on chromosomes 1, 4, 5, 7, 8, and 10 while Zila identified them on chromosomes 1, 4, 5, and 9 [8,44]. Chen et al. [46] found that genes located on chromosome 4 were responsible for fusarium resistance. In a study by Li et al. [47] four QTLs were detected on chromosomes 3, 4, 5 and 6. The resistance allele in each of these four QTLs was attributed to the resistant parent BT-1 and accounted for 2.5–10.2% of phenotypic variation. According to the authors, the QTL with the largest effect detected on chromosome 4 can be treated as a locus of resistance to maize cob fusarium.

In the present study, and as a result of association mapping, 6816 markers significantly associated with fusarium resistance were selected. Among these were both SNP and SilicoDArT markers. In order to narrow down the number of markers for physical mapping, 10 markers that were significant in both localities (Kobierzyce and Smolice) were selected from among all of them. These markers, as in the work of De Jong et al. [45], were located on chromosomes 2, 3, 4, 5, 7, 8 and 10. Physical mapping determined the location of each marker and designed primers for their identification. Markers linked to candidate genes coupled to fusarium resistance can serve to select fusarium-resistant varieties.

Marker-assisted selection (MAS) reduces financial expenses and increases productivity. By increasing the efficiency of selecting varieties for crosses, breeders can improve breeding programs in less time [48]. Salah et al. [49], using MAS, identified genes for resistance (QLT) to *Fusarium* fungi in maize coupled to RAPD (OPA02), ISSR (AD8), SSR (SSR93, SSR105, SSR225 and SSR337) and STS (STS03) markers. The SSR and STS markers were shown to be located on chromosome 10 [49]. The use of SNP markers associated with yield structure traits in maize and barley, showed greater precision than methods based on the study of metabolic pathways [9].

In the present study the analyses revealed that only two markers (11801 and 20607) of the ten tested differentiated between susceptible and resistant genotypes. Two selected markers occur at a higher frequency in resistant genotypes compared with susceptible genotypes. For example, marker 11801 occurs in 8 out of 10 resistant genotypes and 5 out of 10 susceptible genotypes. Importantly, susceptible genotypes S1, S3, S5 and S9 have a field resistance of 6, i.e., they are allowed to contain the 11801 marker that is linked to the candidate gene. Fusarium resistance is a multi-gene trait and therefore single resistance genes can appear in susceptible genotypes with field resistance, such as 6. For the remaining eight markers, monomorphic amplification products with sizes consistent with expected values were observed on agarose gels. Further analysis showed that marker 11801 is within 620 bp of the phosphoenolpyruvate carboxylase kinase 1 gene, which may be involved in resistance processes and can be used to select fusarium-resistant varieties. Additionally, phosphoenolpyruvate carboxylase (PEPC) is a key enzyme involved in several important physiological functions in plants, such as C4 and crassulacean acid metabolism (CAM) photosynthesis as well as the anaplerotic pathway [50].

## 4. Materials and Methods

### 4.1. Plant Material

The plant material includes 66 inbred lines, 122 F1 hybrids and 20 reference genotypes (susceptible and resistant to fusarium). The plant material comes from Plant Breeding Smolice Ltd. IHAR Group in Smolice, Poland (51°42′12″ N 17°10′10″ E) and Malopolska Plant Breeding Sp. z o.o. in Kobierzyce, Poland (50°58′17″ N 16°55′50″ E).

### 4.2. Methods

#### 4.2.1. Field Experiment

During the implementation period of the field experiment, observations were made on the infestation of maize plants by fungi of the genus *Fusarium*. At the stage of the full maturity of maize (BBCH 89), the number of plants with symptoms of fusariosis on the cobs was determined on two dates, every two weeks. The degree of infestation of maize cobs by fungi of the genus *Fusarium*, was evaluated on a 9 point scale [51] (Table 5). The index of cob infestation by *Fusarium* spp. was calculated according to the Towsend–Heuberger formula [52]. The results were converted to Bliss angular degrees and subjected to analysis of variance in this form. In addition, at the wax maturity stage (BBCH 85), the percentage of plants with visible signs of maize caterpillar European corn borer (*Ostrinia nubilalis Hbn*.) feeding on maize cobs was determined.

#### 4.2.2. DNA Isolation

DNA isolation from 66 inbred lines and 122 F1 hybrids that were submitted for next-generation sequencing and 20 reference genotypes was conducted using a reagent kit from Promega. Both isolations were carried out according to the methodology provided with the kits. The concentration and purity of the isolated DNA samples were determined using a DS-11 spectrophotometer from DeNovix. The isolated DNA matrix was brought to a uniform concentration of 100 ng/μL by dilution with distilled water. The efficiency of a single isolation was high and ranged from 106 ng/μL for line 15 to 935.24 ng/μL for line 34. The purity of individual samples was also very good and ranged from 1.7 to 2.0 for absorbance 260/280 and 260/230, respectively.

#### 4.2.3. Genotyping

Genotyping was performed using DArTseq technology, based on next-generation sequencing. The isolated DNA of the tested maize plants, 25 µL at 100 ng from each genotype, was sent in two 96-well Eppendorf plates for analyses aimed at identifying silicoDArT and SNP polymorphisms. The analyses were performed at Diversity Arrays Technology, University of Canberra, Australia. After DNA isolation, the next step was digestion of genomic DNA with restriction enzymes, e.g., Ape KI, Pst I, Msp I, to reduce genome complexity. The use of restriction enzymes for the controlled reduction of genome complexity in combination with NGS was first described by Baird et al. [39]. The original GBS method used a single Ape KI enzyme [43]. Later, the method was expanded to include two enzymes: one rare-cutting Pst I in combination with a second frequent-cutting genomic DNA Msp I [53]. The use of such a version allows the creation of a homogeneous library and enables the detection of most fragments associated with a rare-cutting enzyme. The restriction enzymes used are sensitive to methylation, so it is possible to filter out non-coding regions and methylated repetitive sequences, such as mobile elements. Fragments of genomic DNA cut by restriction enzymes were subjected to ligation with adaptors. Because the latter contain identifiers (barcodes), the origin of each sample was strictly defined, and the identifiers met the relevant criteria [54]. The resulting post-PCR products were analyzed for size and constituted a genomic library, which was then sequenced using the most important platform for NGS, Illumina, according to the methodology described in detail on Diversity Arrays Technology’s website: https://www.diversityarrays.com/technology-and-resources/dartseq/ (accessed on 26 July 2023).

#### 4.2.4. Statistical Analysis

The normality of the distribution of the maize plant infection by *Fusarium* fungi was tested using Shapiro–Wilk’s normality test to check whether the analysis of variance (ANOVA) met the assumption that the ANOVA model residuals followed a normal distribution. The homogeneity of variance was tested using Bartlett’s test. A two-way analysis of variance (ANOVA) was carried out to determine the main effects of genotype, location, and genotype × location interaction on the variability of the degree of maize plant infection by *Fusarium* fungi. The genetic similarity for each pair of the investigated genotypes was estimated based on the coefficient proposed by Nei and Li [55]. The genotypes were grouped hierarchically using the unweighted pair group method of arithmetic means (UPGMA) based on the calculated coefficients [56]. The relationships between the genotypes were presented in the form of a dendrogram [57,58,59]. All analyses were conducted in Genstat 23 [60].

#### 4.2.5. Association Mapping Using GWAS Analysis

Association mapping of 188 maize genotypes (66 lines and 122 hybrids) was performed using GWAS analysis. This mapping was performed based on the results obtained from genotyping and phenotyping. The genotypic data were obtained from DArTseq analysis, while the phenotypic data represent field results on the degree of fusarium head blight. Based on the GWAS analysis performed, silicoDArT and SNP markers with the highest level of significance, i.e., those that were most strongly associated with plant resistance to fusarium, were selected for further study.

#### 4.2.6. Physical Mapping

The silicoDArT and SNP marker sequences selected based on the GWAS analysis were subjected to Basic Local Alignment Search Tool (BLAST) analysis, which involves searching databases to find sequences with high homology to the selected silicoDArT and SNP marker sequences. These analyses were performed in the Unité de Recherche Génomique Info (URGI) service with the completely sequenced maize genome. The URGI program was used to indicate the chromosomal locations of retrieved sequences similar to those analyzed and to determine their physical locations. The sequences of all genes located in the designated area on the chromosome were subjected to further analysis.

#### 4.2.7. Functional Analysis of Gene Sequences

Functional analysis was performed using the Blast2GO program. The sequences of all genes located in the chromosome region designated based on the BLAST analysis performed in the URGI service were analyzed. The goal was to obtain information on the biological function of gene sequences located in the designated chromosome region.

#### 4.2.8. Designing Primers for Identified SilicoDArT and SNP Polymorphisms Related to Fusarium Resistance

The Primer 3 Plus program was used to design the starters.

#### 4.2.9. The Polymerase Chain Reaction (PCR)

Identification of new markers coupled to genes associated with plant resistance to fusarium was carried out using polymerase chain reaction (PCR). The location of the markers and the genes coupled to them are described in the results section.

The polymerase chain reaction (PCR) was conducted in a BIO-RAD T1000 thermocycler. Reagents from Promega were used to prepare the mixture. The basic composition of the reaction mixture is shown in Table 6. This composition was modified depending on the marker being identified.

The conditions for carrying out the PCR reaction were determined individually for each of the identified markers and differed, among other things, in the temperature of attachment of the primers, determined by their melting point. The overall profile of the PCR reaction is shown below:
Pre-denaturation: 5 min at 94 °CPrimer binding: 40 cycles  Denaturation:45 s at 94 °C  Attachment of primers:1 min at X °C  Synthesis:1 min at 72 °CFinal synthesis: 5 min at 72 °CStorage max: 24 h at 4 °C

#### 4.2.10. Electrophoresis

Electrophoresis of PCR reaction products was carried out in a 1.5% agarose gel, with the addition of 1 µL of Midori Green solution, for 2 h at 100 V. The O’RangeRuler 100 bp bar size marker from Fermentas was used to identify molecular masses. Visualization of the separated DNA fragments was performed using UV light and recorded on digital images using the BIORAD gel visualization and documentation system.

## 5. Conclusions

Excessive use of chemical plant protection products negatively affects the natural environment. Pesticides also pose a direct threat to human health and sometimes even life. To prevent public exposure to widespread environmental contamination, integrated pest management methods should be introduced. The general principles of integrated pest management are to prevent the occurrence of pest organisms or to reduce their occurrence and negative impact, which can be achieved, among other things, by using resistant or tolerant varieties. As a result of field experiments and in-depth molecular analyses, molecular markers conjugated with potential genes for resistance to maize ear fusariosis were selected. The newly selected markers were tested against reference genotypes. As a result of the analyses, it was found that 2 markers (11801 and 20607) out of 10 tested differentiated between susceptible and resistant genotypes. Marker number 11,801 proved to be the most effective, with a specious product of 237 bp appearing for genotypes 1, 3, 5, 9 and 10. These genotypes were characterized by a field resistance of 4–6 on the 9° scale (1 being susceptible and 9 being resistant) and for all genotypes except 16 and 20, which were characterized by a field resistance of 9. Plant resistance to fusarium is a multigenic trait and the selected markers can be used to select genotypes together with markers of other genes involved in the immune reaction.

## Figures and Tables

**Table 1:** Analysis of variance for maize plant infection by *Fusarium* fungi.

Source of Variation	Degrees of Freedom	Sum of Squares	Mean Square	*F*-Statistics
Genotype (G)	187	252.2163	1.3416	3.90 ***
Location (L)	1	22.9549	22.9549	66.66 ***
G × L interaction	187	156.934	0.8348	2.42 ***
Residual	484	166.6667	0.3444	

*** *p* < 0.001.

**Table 2:** SilicoDArT and SNP molecular markers significantly associated with maize plant resistance to fusarium in Kobierzyce and Smolice (significant associations selected at *p* < 0.001 with correction for Benjamini–Hochberg multiple testing).

		Number of Identified SilicoDArT and SNPs Markers Associated with Fusarium Resistance in Maize	
		Kobierzyce	Smolice
Number of significant markers	DArT	4885	390
	SNP	1100	141
	Total	5985	531
Minimal effect	DArT	−0.431	−0.68
	SNP	−0.425	−0.64
	Total	−0.431	−0.68
Maximal effect	DArT	0.573	0.67
	SNP	0.566	0.601
	Total	0.573	0.67
Medium effect	DArT	0.371	0.009
	SNP	0.362	−0.041
	Total	0.369	−0.005
Total effect	DArT	1810	3.395
	SNP	398	−5.84
	Total	2208	−2.44
**Percentage of variation explained**			
Min	DArT	5.2	5.2
	SNP	5.2	5.2
	Total	5.2	5.2
Max	DArT	20.8	12.2
	SNP	21.2	11.4
	Total	21.2	12.2
Medium	DArT	10.73	6.48
	SNP	10.15	6.57
	Total	10.62	6.50

**Table 3:** Characteristics and location of markers significantly related to plant resistance to fusarium (significant associations selected at *p* < 0.001 with correction for Benjamini–Hochberg multiple testing).

Marker	Marker Type	Chromosome	Marker Location	Candidate Genes
27364	SilicoDArT	Chr 4	2451800	95,397 bp at 5′ side: at-hook motif nuclear-localized protein 23 5235 bp at 3′ side: uncharacterized protein loc100286026
26864	SilicoDArT	Chr 2	2583292	765,204 bp at 5′ side: germin-like protein subfamily 1 member 17 precursor, 85,363 bp at 3′ side: dna-directed rna polymerases ii, iv and v subunit 9a
17777	SilicoDArT	Chr 3	4769959	uncharacterized protein loc100275570 isoform X1, uncharacterized protein loc100275570 isoform X1
27435	SilicoDArT	Chr 10	4770049	6549 bp at 5′ side: starch synthase iiib-1 precurs or 8366 bp at 3′ side: uncharacterized protein loc103642070
27775	SilicoDArT	Chr 3	25946459	uncharacterized protein loc100501166
11801	SilicoDArT	Chr 5	4764756	620 bp at 5′ side: phosphoenolpyruvate carboxylase kinase 1, 11,629 bp at 3′ side: uncharacterized protein loc103628906 isoform X1
24753	SilicoDArT	Chr 8	2440301	33,881 bp at 5′ side: uncharacterized protein loc100281900, 14,700 bp at 3′ side: uncharacterized protein loc103636139
20607	SilicoDArT	Chr 7	24015383	210 bp at 5′ side: uncharacterized protein loc100383808 isoform X2, 7460 bp at 3′ side: uncharacterized protein loc100272692
73234	SNP	Chr 2	34766405	putative uncharacterized protein ddb_g0274535
8596	SilicoDArT	Chr 7	4592527	116,269 bp at 5′ side: uncharacterized protein loc10019402712714 bp at 3′ side: putative cytochrome p450 superfamily protein

**Table 4:** Sequences of designed primers used to identify newly selected markers significantly associated with plant fusarium resistance.

Marker	Primer Sequences		Melting Temperature (°C)	Product Size (bp)
	Forward	Reverse		
27364	AAGACTGGGGGTAGCTGCAG	CTTGACCGGTAGCGATTGGA	62	269
26864	GCACGCTGGATGTTGCAG	GACGGGCATGTGATCTAACGA	65	138
17777	ACCAGAAGAACATTCTGCAG	GAACGAGCTCACTCAGAAGC	57.5	167
27435	AAGGCGTGCTCCATCTGCAG	CGGTCACCACTCACCAGGTA	63	333
27775	AGAATTGAGATCCTCTGCAG	TTGGTTTTCCATTTTCCCGC	62	273
11801	GCAACGTGGCGTCTCTGCAG	ATGCTGATACGGTTGGAGTCAGT	59	237
24753	AGCTAGCTTTGGTCCTGCAG	TCAAAGGCGAACGTAGCGAT	65	336
20607	TCACTTTTTCAGTTCTGCAG	AACGAACCAAACAAGCCTTA	60	137
73234	ACTATAAAGGAACTCCTGCAG	TCTACTGTAGCAGGAATGGGA	59	486
8596	TTCTAGGGTGTCACCTGCAG	GCCCTTAGTCTAAAGCGGCA	64	310

**Table 5:** Interpretation of the scale for assessing the colonization of cobs by fungi of the genus *Fusarium*.

Degree	Description
1	pure ear, without mycelium
3	single fungal colonies on the ear
5	50% of ear colonized by mycelium
7	75% of ear colonized by mycelium
9	the whole ear colonized by mycelium

**Table 6:** PCR reaction mixture composition.

Stock Solution and Components	Per Reaction	Per 1 Sample (20 µL)
Buffer (5× Green Go Taq, Flexi Buffer)	1X	4
25 mM MgCl_2_	1.5 mM	1.6
10 mM Ultrapure dNTPs Mix	0.1 mM	0.32
DNA polymerase (Go Taq G2 Flexi)	0.05 U/µL	0.17
Primer I FW	0.3 µM	0.25
Primer II RW	0.3 µM	0.25
Nuclease-free water	-	11.91
DNA	0.05 µg to 1 µg	1

## Data Availability

The data presented in this study are available on request from the corresponding author.
